# The UK Infected Blood Inquiry: A Personal Reflection

**DOI:** 10.1111/hae.70043

**Published:** 2025-04-02

**Authors:** Christine A. Lee

**Affiliations:** ^1^ University College London London UK

**Keywords:** haemophilia, HCV, HIV, infected blood inquiry

## Abstract

The UK Infected Blood Inquiry (IBI) considered the transfusion transmitted infections hepatitis B and C, HIV, and vCJD. The inquiry lasted 6 years during 2018 to 2024 and reported to the UK parliament on 20 May, 2024. It concluded that at least 3000 people died because of the recklessness and obstinacy of officialdom and it has been reported that victims may be compensated an estimated £12 bn–£14 bn ($16 bn–$18 bn). This personal reflection considers people with the bleeding disorder haemophilia, who were infected with blood products. The longevity of both the understanding of the infections, as well as the long natural history of the infections themselves, is set in the context of the medicine practiced by physicians with whom the author was taught and practiced over a period of 40 years. These physicians used blood products to advance the care and life expectancy of patients whilst the ill understood and unforeseen side effects of treatment were slowly elucidated: their contributions were severely criticised but their evidence could not be presented through ill health or death. It is recommended that ‘no‐fault compensation’ schemes should be provided, especially in the era of new and sometimes irreversible therapies, and evidence‐based medicine is protected.

## 1 Introduction

The UK Infected Blood Inquiry (IBI) represented a collision between government and medicine which was adjudicated by the law. The Times commented [1] ‘Inquiries themselves have a perverse tendency to frustrate the very objectives they are designed to meet, namely, the swift determination of the crucial facts and the conferral of corrective justice’. The inquiry took 6 years from 2018 to 2024 at a cost of roughly £30 m ($40 m); it concluded that at least 3000 people died, in large part because of the recklessness and obstinacy of officialdom and may compensate victims £12 bn–14 bn ($16 bn–18 bn) [2]. It has also been reported that a person infected with HIV can expect between £2.2 and £2.6 million ($3 m–3.5 m) and those with a chronic hepatitis C infection £610,000 to £810,000 ($0.8–$1) in compensation [3].

The Report of the IBI was presented to the UK parliament (pursuant to section 26 of the Inquiries Act 2005) on 20 May, 2024. The principal infections considered by the inquiry were hepatitis (B and C) and HIV and the transmission of vCJD was ‘also considered’ [4]. The IBI considered people with bleeding disorders, who were infected predominantly by blood products, and those infected by blood transfusions (rather than blood products) since the inception of the National Health Service in 1948.

The inquiry was judge led and conducted in an adversarial style. There was little contemporaneous medical input because many of the relevant clinicians were dead or too ill to be subjected to interrogation. The enormous contribution made by such physicians to enable a good quality and expectancy of life in a previously lethal rare inherited disorder was therefore unrecognised. The clinical practice of these physicians was severely criticised and described as ‘research’ with little understanding of evidence‐based medicine. There was little consideration of the consequences of no treatment and the clinical reason for treatment: the risk benefit analysis. International medical experience was not explored. The long time‐scale of the evolution and communication of knowledge, as well as the infections themselves, created a complexity which was a challenge for those trained in law and even for many contemporary physicians.

## 2 A Personal Reflection

In 2013, as editor of *Haemophilia*, I co‐edited a commemorative issue to celebrate 50 years of WFH, which contained a compilation of historical papers [5]. It contained the following quotation:

*‘The history of haemophilia shows the human mind attempting to define and encompass a mysterious yet fascinating phenomenon; and the human heart responding to the challenge of repeated adversity’* [6]

This is a very personal reflection: I want to consider the IBI in the context of my personal experience in the field of haemophilia and transfusion transmitted disease. I hope to explain the contribution made by physicians who were my mentors, by whom I was taught, and with whom I worked throughout my medical career during the years 1962 and 2008, and who have now died: Paul Beeson, Janet Vaughan, Rosemary Biggs, Katharine Dormandy, Charles Rizza and Peter Kernoff: all were physicians who certainly helped to ‘define’ haemophilia and respond to ‘the challenge of repeated adversity’. I believe that it is important to document the past history of their evidence‐based medicine in order that misunderstandings within the IBI can be corrected.

## 3 Post Transfusion Hepatitis

The IBI concluded that clinicians and the national health service were ‘*complacent about the risks of non‐A non‐B hepatitis (HCV)*’, failed ‘*to tell people of the risks of transfusion*’ and delayed ‘*informing people of their infections by weeks, months and sometimes years*’ [7]. The evolution of knowledge about transfusion and HCV was slow: compounded by the late genetic identification of the virus only in 1989 [8], enabling an antibody test available for routine use in 1991, and the identification of circulating virus by PCR in 1992.

As a medical student in Oxford in 1966, I was taught by Paul Beeson, the Nuffield Professor of Medicine, who had authored a paper in 1943 describing clinical jaundice occurring after transfusion: this was most likely HCV [9]. In the conclusion, he writes that a careful record should be made of the source of the blood or plasma and ‘a small portion of blood or plasma should be set aside at the time the transfusion is given, so that in the event of subsequent cases of hepatitis, some of the causative material will be available for study’. In 2024, the IBI criticised clinicians for ‘*testing samples (which in most cases patients did not know were to be retained for the purpose) without their knowledge and consent’* [10].

I had studied animal physiology as an undergraduate at Somerville College, Oxford where Janet Vaughan was principal; a haematologist, she had led the N.W. London blood transfusion service during 1940–45 (WWII) and had attempted to follow up patients, most of whom were air raid casualties treated under emergency conditions, who had been transfused with serum or plasma in 78 hospitals. The aim of this study was to determine the incidence of homologous serum jaundice following transfusion, its incubation period, and symptomatology [11]. This study is significant because it considers risk and benefit: *‘it appeared important to determine, if possible, the incidence of this complication, since it might well be that the risk of hepatic necrosis was greater than the risk incurred by with‐holding transfusion.’* The results showed that the incidence of jaundice was 7.3% in 1284 patients who received pooled serum and/or plasma, but there was no jaundice in 1284 who received whole blood. It was concluded that for transfusion purposes, only small pools should be used. However, the majority of the patients given pooled plasma were civilian air‐raid casualties and clearly in the risk benefit analysis, the treatment of the crush injury was more important than consideration of the risk of jaundice.

## 4 Lord Mayor Treloar School

The IBI report was very critical of ‘*research*’, particularly in the context of The Lord Mayor Treloar school (Treloar), which began to accept a large number of boarders with haemophilia in the 1960s. The IBI used written comments to suggest that a group of boys were brought together specifically to perform research: ‘*nearly 40 haemophiliacs in the College’* provided ‘*an opportunity for research’* and this was encouraged by ‘*the very valuable help of Dr Rosemary Biggs and Dr K.M. Dormandy’*. There are unqualified statements attributed to Rosemary Biggs: ‘*the collection of 49 haemophilic patients at the Alton School makes this a unique opportunity to study the disease*’ and that a major research objective was to define ‘*the essential organisation required to treat boys gathered in one school’* [12]. Rosemary Biggs is described, quite rightly, as a central figure in haemophilia at that time.

In 19966, Rosemary Biggs, aged 86, was interviewed in her home by Charles Rizza and myself for the Wellcome Witnesses to the Twentieth Century Medicine series [13]. The following is an extract from that interview which describes in her own words the difficult and poor life expectancy of children in the 1960s and the co‐operation with Treloar:

*‘Prof. Lee: When I was a medical student at Oxford I have one memory of 1967, when we did orthopaedics, which was a ward of children, of small boys. Tell us a bit about boys at that time*.

*Dr Biggs: They all went to the Nuffield Orthopaedic Centre which had developed a quite considerable expertise. They didn't have a lot of treatment. They had very slow careful manipulation of joints to get them back into approximately a good position and the best result we ever hoped for was that they would fix in a position of reasonable usefulness for arms and legs so they could raise your arm in a lesson, and stand on a useful leg. Of course, it was at this time the whole situation changed, because when we started treatment most of the patients didn't live to be grown up. The average age of death was 16, so there wasn't much purpose in giving education a top priority. Once patients were going to live for a reasonable length of time they had to be educated, and well‐educated, because they would never be able to do manual work. When they went to the Nuffield Orthopaedic Centre they used to get lessons and then the question arose: should further steps be taken in this direction? We co‐operated with the Lord Mayor Treloar College at Alton, and they took haemophilic pupils’*

Biggs and Dormandy, both experienced physicians caring for haemophilia boys at the time, had conducted a survey of the parents of boys with haemophilia, which made the case for establishing a boarding‐school for haemophilic boys to be run in association with a haemophilia treatment centre in order that boys could be both educated and treated [14]. A historical annotation of this study published in 1997 [15] commented:

‘The Haemophilic Boy in School’ was an extraordinary paper for its time because the authors looked at quality of life rather than just the coagulation disorder. The emphasis on the recognition of disease and its treatment that prevailed is summed up in the first sentence, Education is not a subject that would normally be discussed in a medical journal, but… At the time Rosemary Biggs was with her colleague R.G. MacFarlane spearheading the basic research that would result in effective therapy’.

These physicians watched effective treatment for haemophilia emerge and documented best practice in a group of children with a previously lethal rare disorder. The poor life expectancy of 16 years meant there were few adult patients alive.

However, the IBI was extreme in their criticism:

*‘Infections, leading to deaths, illness and suffering were caused needlessly to* ***people with bleeding disorders*** *by*:

*Treating children at Treloar's with multiple, riskier commercial, concentrates prophylactically and objects of research*.

*Treating children unnecessarily with concentrates (especially commercial concentrates) rather than choosing safer treatments* [16]

The rationale for prophylaxis, which had resulted in large quantities of concentrate, much derived from US donors infected with the yet to be identified HIV, was condemned as unwarranted ‘research’. The IBI did not consider international practice in Europe and North America, where there were reports of small groups of boys on prophylaxis in the 1960s and 70s; in Europe, it was evident in several countries as early as the 1970s, or even earlier, that prophylaxis was state‐of‐the‐art treatment, at least in children [17]. The difficulty of providing evidence in a rare disorder required the observation of a group of boys with haemophilia and this was the opportunity Rosemary Biggs encouraged. In the face of world‐wide concern about the cost of the increased concentrate requirement, a double‐blind trial to prove efficacy and justify the funding was possible in the United Kingdom [18]. The IBI severely and unfairly criticised Aronstam, the physician who conducted this trial because many boys, unknowingly, became infected with HIV.

Rosemary Biggs had highlighted the natural history of haemophilia without treatment in her study charting 30 years of haemophilia treatment in Oxford published in 1967 [19]. A monograph published by Carroll Birch in 1937 [20] in the USA was summarised: the cause of death in 113 patients showed that many died from very trivial injury; 82 died before 15 years of age and only eight survived beyond 40 years. Within the IBI, the voluminous criticism of both Biggs and Aronstam largely ignores the lethal outlook for haemophilic individuals without treatment, and there is little consideration of contemporary international, particularly European, research experience of prophylaxis.

## 5 First Treatment With FIX Clotting Factor Concentrate

Throughout the IBI report there is much criticism of the treatment of children without consent. In 1959 Charles Rizza came from Scotland as a clinical research fellow under Biggs at Oxford where the MRC Blood Coagulation Research Unit was supporting the early development of clotting factor concentrates. In Scotland, a 4‐year‐old child, with severe factor IX deficiency, had developed a large haematoma following venepuncture, which caused osteomyelitis of the radius and ulna and necrosis of the lower arm. Rizza knew that Ethel Bidwell had successfully prepared FIX concentrate at Oxford but it had never been used. The child was flown to Oxford and the factor IX concentrate was given to the child for the first time to cover an arm amputation. This saved his life as recounted in the historical annotation published in 1995 [21, 22]. Rizza was too ill to give evidence at the inquiry and has since died: his unique experience would have informed the IBI of the early development NHS clotting factor concentrates and their transformative effect on the lives of patients.

## 6 The Emergence of AIDS and Early Studies of Non‐A Non‐B Hepatitis

In the early 1980s, the only available tests for assessing immune deficiency were T cell subsets, the T4/T8 ratio. It was recognised that some groups of haemophilic patients had normal ratios but many showed immunosuppression. There was great debate as to whether this was due to the donor pool or the manufacturing process of the clotting factor concentrates. We reported normal ratios in FIX‐deficient patients and, in retrospect, wrongly concluded ‘that low T4/T8 ratios may have simple biochemical causes and should not necessarily be regarded as being predictive of AIDS’ [23]. The idea of a prodromal or ‘forme fruste’ of AIDS was debated. We tried to elucidate this by comparing T4/T8 ratios in patients treated with both NHS and commercial concentrates at the Royal Free with those treated at Oxford where there was a long tradition of making concentrates from NHS plasma. For the IBI, the main consideration was that Rizza had tested children without parental consent and that patients had not been told they would develop AIDS. I wrote to Rizza ‘I do have a nasty feeling that NHS concentrate is going to turn out safer’: this was the beginning of a recognition that the NHS donor pool was safer and we had been previously wrong attributing the immune suppression to the fractionation process. This illustrated our confusion and lack of understanding before an HIV test became available in 1984–45 [24, 25]. It is unfortunate that Rizza was too ill to set this in context and was not questioned.

In the United Kingdom, Katharine Dormandy had pioneered the manufacture of cryoprecipitate in the Department of Haematology at the Royal Free Hospital which made home treatment for haemophilia possible [26]. Much of the criticism of the IBI was that cryoprecipitate was an effective alternative to concentrate. Patients at the Royal Free were treated exclusively with cryoprecipitate during the 1970s and therefore, in 1977, Dormandy was able to compare transaminases in her patients to those in patients treated exclusively with the new concentrates in Worcester, the United States. Both groups showed abnormal transaminases, but this was more common in those treated with concentrates in the United States and it was noted: ‘The long‐term consequences of the various abnormalities recorded here is unknown. Aggressive therapy should continue’ [27]. In retrospect, the raised transaminases showed HCV infection: the estimated carriage rate was 3.3/1000 in the United Kingdom [28] and therefore infection followed both large exposure to cryoprecipitate in the United Kingdom and large pool concentrates in the United States.

Peter Kernoff trained under Rizza at Oxford where he completed a PhD researching inhibitors in haemophilia. Following the death of Katharine Dormandy in 1977, Kernoff became director of the Royal Free haemophilia centre, and in order to accurately record the treatment records of the large number of patients with inherited bleeding disorders who attended the centre, one of the largest in Europe, a ‘computer expert’ was employed in the early 1980s. A protocol to store patient blood samples was established primarily for surveillance of inhibitor development, but also available when it was hoped there would be a test for hepatitis.

In 1983, I was funded by the charity Action Research for the Crippled Child to document the clinical details of 58 patients who had been treated between 1978 to 1983 and had received a first exposure of large‐pool clotting factor. The diagnosis of non‐A non‐B hepatitis (HCV) was a challenge because of the long incubation period, it was clinically asymptomatic in the majority of patients, the transaminases were only intermittently abnormal and there were no markers. Diagnosis of non‐A non‐B infection required a patient to have a normal transaminase before first treatment with concentrate, and an elevation of transaminase 2.5 times the upper limit of normal during follow‐up every 2 weeks for 3 months. Markers enabled exclusion of hepatitis A, B, EBV, and CMV. In a pivotal study, it was shown that there was a virtual 100% attack rate irrespective of whether the donor plasma was NHS‐ or US‐derived. This study was published [29] and contained within my dissertation for MD [30] Significantly, Kernoff considered that the findings of this study would be a useful basis for comparative studies for the evaluation of new products with possible reduced infectivity obviating the need for controls.

In my evidence to the IBI, I had stated that the study was ‘*retrospective in the sense these people were not recruited to go into a study. They were people who came in with a bleeding problem, and then retrospectively identified. And because there had been a collection of the samples and the results of liver function tests, it was possible to retrospectively analyse that information’* [31]. The judge wrote ‘*I reject this description, which is not consistent in any event with the report of the study that was published, and which would not explain why so many participants were treated with concentrates when a different treatment would have been an obvious and safer choice.’* [31]

The variety and severity of bleeding disorders and the reasons for treatment were complex but this was not considered by the judge. For medical reasons, Kernoff was unable to work from 1991 and he died in 2006. I conducted the data collation and analysis in close collaboration with Kernoff but my evidence was refuted and I was not given an opportunity to correct the report prior to publication. Kernoff made a major contribution to the understanding of non‐A non‐B hepatitis in haemophilia in the early 1980s, but the IBI was unfairly critical: in particular the suggestion that four children within the study were ‘experimented’ upon was wrong.

## 7 The Natural History of HIV

The IBI report considered that people with bleeding disorders were failed by clinicians and the health service by *‘Testing samples (which in most cases patients did not know were to be retained for the purpose) and without their knowledge or consent’* [31]. This was written in the context of the modern practice of medicine, which requires the physician to obtain written informed consent for the storage and future use of unused samples. Guidance and a template for informed consent has been published by the Research and Ethics Committee of WHO (WHO ERC) [32].

In 1978, Kernoff, as director of the Royal Free Haemophilia Centre, had set up a protocol for the care of patients with haemophilia: patients with severe disease were clinically reviewed every 6 months and those with non‐severe haemophilia every year. At each visit, for review or treatment, a plasma sample was retained. At this time, formal written consent was not required, but the regular reviews provided an opportunity for the physician to share information and discussion with the patient about the blood tests performed.

In 1985, a test for HIV became available, and 112 patients who were receiving care for their haemophilia were found to be HIV positive. These results were given to the patient in person by the consultant physician, often together with the family therapist.

In retrospect, using stored specimens, it was possible to show that HIV seroconversion occurred during the years 1980 to 1985 [33], and that more than half of the infections had occurred before 1982 when the first cases of AIDS (*Pneumocystis carinii* pneumonia in three people with haemophilia) were reported by the Morbidity and Mortality Weekly Report (MMWR) [34]. Availability of a test for HIV in 1985 was coincident with the first heated concentrates which destroyed HIV, and no further seroconversions occurred after 1985. This retrospective information was first published in 1989 and informed the understanding and management of not only haemophilic patients but others who were infected with HIV. The study showed that the risk of developing AIDS was negligible until the T4 lymphocyte count fell below 0.2 × 10the 9 should be a superscript /L and therefore was an indicator of the patient's condition and informed treatment for immune suppression. This information was slowly accrued and dependent on careful clinical observation and the initial testing of stored plasma samples to establish the time of seroconversion in a unique group of patients. The IBI did not appreciate the slow evolution of knowledge and the understanding of HIV infection and the retrospective vision failed to understand the confusion and knowledge available during the years 1980 to 1985. We attempted to provide a detailed description of HIV‐related disease which would provide a useful control for future drug trials in other asymptomatic at‐risk groups.

The chair of the IBI, Sir Brian Langstaff, was required to send a warning letter to any person he considered may be subject to explicit or significant criticism in his final report. The record of the criticism I received is illustrative of the poor state of our knowledge in 1984, before HIV had been isolated.

In a ‘fact sheet’ published by the UK Haemophilia Society in May 1984, I had written: ‘In Great Britain the number of haemophiliacs who have been reported with AIDS remain at 2. Thus the incidence is less than 1 in 1000 patients at risk.’ (35) The chair considered: *‘The paper conflated the words ‘risk’ with ‘incidence’. Dr Lee's words were either a meaningless addition or a muddled expression.’* In 1984, the UK national records showed 2000 patients with haemophilia had been treated with large pool clotting factors and were therefore ‘at risk’ and only two patients had the clinical syndrome of AIDS as defined by MMWR [34]: thus the incidence was less than one in 1000 patients at risk. This shows the paucity of the knowledge and understanding in 1984, and also the difficulty for those trained in law to interpret clinical facts [35].

## 8 Conclusions

In 1986, Kernoff and I compiled and wrote in the introduction to a collection of historic papers entitled Blood Product Therapy in Haemophilia ‘that effective treatment is rarely free from adverse effects’ and that the field of haemophilia ‘provides one of the best examples in medicine of how basic science and clinical research can, in a very short time, lead to advances in clinical practice’ [37]. Effective treatment has transformed both the quality and expectancy of life for people with haemophilia, but the treatment given before 1985 has resulted in the devastating side effect of transfusion transmitted disease. A well‐documented chronology of AIDS, hepatitis and haemophilia reflects on ‘the fallacy of retrospective knowledge’ where the past is judged with the perspective of present knowledge [38]. The failure of the IBI has been to interpret the chronology and the complexity of transfusion transmitted disease almost half a century after the physicians, who tried to elucidate the problems in order to deliver best practice, have died. In my oral evidence to the IBI, I said I did not like compensation because it suggests liability [39].

I strongly believe a ‘no‐fault compensation’ scheme should be established, especially in the current era of new and sometimes irreversible therapies [40], in order that adequate compensation and support can be provided for any injured person. Scientific enquiry of innovative treatments is essential and there should not be victimisation if unforeseen side effects occur.

## Author Contributions

Written entirely by Christine A. Lee.

## Conflicts of Interest

The author declares no conflicts of interest.

## Data Availability

Data sharing is not applicable to this article as no new data were created or analysed in this study.
